# *GNAS* mutations suppress cell invasion by activating MEG3 in growth hormone–secreting pituitary adenoma

**DOI:** 10.32604/or.2024.046007

**Published:** 2024-05-23

**Authors:** CHAO TANG, CHUNYU ZHONG, JUNHAO ZHU, FENG YUAN, JIN YANG, YONG XU, CHIYUAN MA

**Affiliations:** 1 Department of Neurosurgery, Affiliated Jinling Hospital, Medical School of Nanjing University, Nanjing, 210002, China; 2 Department of Neurosurgery, Children’s Hospital of Nanjing Medical University, Nanjing, 210019, China; 3 Affiliated Eye Hospital, Nanjing Medical University, Nanjing, 210029, China; 4 Department of Neurosurgery, Jinling Hospital, Southern Medical University, Nanjing, 210002, China; 5 Department of Neurosurgery, Jinling Hospital, Nanjing Medical University, Nanjing, 210002, China

**Keywords:** GHPAs, GNAS mutation, MEG3, β-catenin, EMT

## Abstract

Approximately 30%–40% of growth hormone–secreting pituitary adenomas (GHPAs) harbor somatic activating mutations in *GNAS* (α subunit of stimulatory G protein). Mutations in *GNAS* are associated with clinical features of smaller and less invasive tumors. However, the role of *GNAS* mutations in the invasiveness of GHPAs is unclear. *GNAS* mutations were detected in GHPAs using a standard polymerase chain reaction (PCR) sequencing procedure. The expression of mutation-associated maternally expressed gene 3 (*MEG3*) was evaluated with RT-qPCR. *MEG3* was manipulated in GH3 cells using a lentiviral expression system. Cell invasion ability was measured using a Transwell assay, and epithelial–mesenchymal transition (EMT)-associated proteins were quantified by immunofluorescence and western blotting. Finally, a tumor cell xenograft mouse model was used to verify the effect of *MEG3* on tumor growth and invasiveness. The invasiveness of GHPAs was significantly decreased in mice with mutated *GNAS* compared with that in mice with wild-type *GNAS*. Consistently, the invasiveness of mutant *GNAS*-expressing GH3 cells decreased. *MEG3* is uniquely expressed at high levels in GHPAs harboring mutated *GNAS*. Accordingly, *MEG3* upregulation inhibited tumor cell invasion, and conversely, *MEG3* downregulation increased tumor cell invasion. Mechanistically, *GNAS* mutations inhibit EMT in GHPAs. *MEG3* in mutated *GNAS* cells prevented cell invasion through the inactivation of the Wnt/β-catenin signaling pathway, which was further validated *in vivo*. Our data suggest that *GNAS* mutations may suppress cell invasion in GHPAs by regulating EMT through the activation of the *MEG3/Wnt/β-catenin* signaling pathway.

## Introduction

Growth hormone–secreting pituitary adenoma (GHPA) accounts for 12.5% of pituitary neuroendocrine tumors, and excessive growth hormone results in acromegaly and systemic complications [1]. Acromegaly has been associated with a two-fold increase in mortality, mainly due to cardiovascular disease, which can be reversed by treatments for controlling hormone overproduction [2]. Approximately one-half of the patients with GHPA experienced high-risk relapse following surgical reduction owing to tumor cell infiltration of the surrounding tissues [3]. Thus, supplementary chemotherapy is warranted to control tumor recurrence.

The genome-wide analysis of epigenetic changes revealed specific subgroup molecular characteristics of pituitary adenoma [4]. For example, the hypomethylation of *GH1* and *SST5* promoters is associated with the overexpression of the respective genes in GHPAs, and *POMC* is hypomethylated in corticotroph adenomas. The somatic mutations of *GNAS* and *USP8* combined with transcriptome analysis have been identified in GHPAs and corticotroph adenomas.

Mutations in *GNAS*, which encodes the α subunit of the stimulatory G protein, were detected in ~40% GHPAs [5,6]. Functional studies have suggested that *GNAS* mutations constitutively activate adenylyl cyclase (AC), thereby inducing the cyclic AMP (cAMP) signaling pathway in pituitary tumors [7]. Studies have reported that somatostatin and its analogs reduce GH secretion through multiple mechanisms involving AC inhibition [8]. Somatostatin binding sites are negatively coupled to AC; however, the inhibition of hormone secretion by somatostatin cannot be explained solely through AC inhibition [8]. It has been speculated that somatostatin blocks basal hormone release by a mechanism different from that of the inhibition of cAMP formation. In addition, it has been suggested that the volume of *GNAS*-mutant GHPA is smaller and it is less likely to be invasive [9].

Maternally expressed gene 3 (*MEG3*), a large non-coding RNA (lncRNA), was first identified as a tumor suppressor in the pituitary [10]. Our previous study suggested that *MEG3* expression is associated with *GNAS* mutations and it is involved in regulation of growth hormone hypersecretion, proliferation, and invasiveness of GHPAs [11]. The cAMP response element (CRE), located in the *MEG3* proximal promoter region, is critical for *MEG3* expression [12]. Cyclic adenosine monophosphate‑responsive element-binding protein, as a downstream target of mutated *GNAS*, is also implicated in the upregulation of *MEG3* by binding to the CRE site [12,13]. Some studies have suggested that *MEG3* suppresses tumor cell proliferation and invasion by downregulating related proteins [14,15]. The Wnt/β-catenin pathway plays an important role in tumorigenesis, cellular proliferation, and invasion [16]. Extensive studies have shown that lncRNAs have the potential to target canonical Wnt-related components during epithelial–mesenchymal transition (EMT) process [17]. Studies have demonstrated that *MEG3* negatively regulates the Wnt/β-catenin signaling pathway in tumor growth and invasion [18,19]. Recently, *MEG3* was shown to induce the phosphorylation, ubiquitination, and degradation of β-catenin through GSK-3β, which in turn inactivates the Wnt pathway and ultimately inhibits the invasion and metastasis of retinoblastoma cells [20]. This study aimed to ascertain whether mutations in *GNAS* inhibit the invasiveness of GHPA cells mainly through *MEG3*-mediated inactivation of the Wnt/β-catenin pathway. The findings of this study may provide a new therapeutic approach for treating GHPA.

## Materials and Methods

### Patients and clinical characteristics

Tumor samples were collected from 44 patients (21 men and 23 women) with acromegaly who underwent endoscopic endonasal transsphenoidal surgery at the Department of Neurosurgery of Nanjing Jinling Hospital (Nanjing, China) between November 2018 and November 2019. The inclusion criteria were as follows: (i) available results of biochemical tests and magnetic resonance images (MRIs) before any treatment, (ii) pituitary adenomas that can be identified via radiological study, and (iii) histological diagnosis of GHPAs by pathologists in Nanjing Jinling Hospital. The exclusion criteria were as follows: (i) patients who have undergone radiotherapy and drug therapy before surgery and (ii) patients with residual or recurrent tumor following surgery. Overall, 10 patients with clinically nonfunctioning pituitary adenoma (NFPA) were included as negative controls. Approval for the study was obtained from the Ethical Committee of Nanjing Jinling Hospital (2018NZKY-008-02) and informed consent were obtained from all patients who participated in this study. This study was performed according to the ethical standards of the 1964 Helsinki Declaration. Pituitary adenomas were classified into invasive and noninvasive tumors according to the degree of lateral extension to the cavernous sinus space via MRI scanning [21]. Knosp grades 3 and 4 were defined as invasive pituitary adenomas, and Knosp grades 0–2 were defined as noninvasive tumors [22]. Tumor volume was determined using the formula (length × width × height × Π)/6. The clinical characteristics of the patients are described in Table 1.

**Table 1:** Comparison of clinical characteristics of patients with and without *GNAS* mutations

	GNAS-MUT	GNAS-WT	*p* value
Patients (n)	16 (36.4%)	28 (63.6%)	
Age (yr)	49.7 ± 9.7	47.5 ± 10.1	0.155
Sex (men/women)	10/6	11/17	0.138
Knosp grade (%)			0.001
0–2	81.3% (13/16)	28.6% (8/28)	
3–4	18.7% (3/16)	71.4% (20/28)	

### Detection of mutations in *GNAS*

Genomic DNA was extracted from 44 GHPA and 10 NFPA tissues using a DNA miniprep kit, according to the manufacturer’s protocol (Qiagen GmbH, Hilden, Germany). Point mutations in *GNAS* reported in tumor specimens included the CGT-to-TGT mutation at codon 201 (Arg201Cys) and the CAG-to-CTG mutation at codon 227 (Gln227Leu) [23]. PCR amplification of codons 201 and 227 was performed using Taq DNA-Polymerase (TTH Biotools Madrid, Spain) [23]. The PCR products were purified using a PCR purification kit (Qiagen GmbH, Hilden, Germany) and sequenced using an ABI3730XL analyzer (Applied Biosystems; Thermo Fisher Scientific, Inc., Carlsbad, CA, USA). Primer sequences used for PCR and DNA sequencing are listed in Table 2.

**Table 2:** Primer sequences for PCR and DNA sequencing

Gene	Primer sequences
*GNAS* codon 227	F: ATCATGGTTTCTTGACATTCACCCC
	R: CCACCACGAAGATGATGGCAGTC
GNAS codon 201	F: CAAGCAGGCTGACTATGTGCCGA
	R: GCTGGCCACCCACGTCAAAC
*MEG3*	F: ATCATCCGTCCACCTCCTTGTCTTC
	R: GTATGAGCATAGCAAAGGTCAGGGC
β-actin	F: CACCCAGCACAATGAAGATCAAGAT
	R: CCAGTTTTTAAATCCTGAGTCAAGC

### Cell culture and transduction

The GH3 cell line (rat GH-secreting pituitary tumor cell line), which produces both growth hormone and prolactin, was purchased from the Cell Culture Centre, Institute of Basic Medical Sciences, Chinese Academy of Medical Sciences (Beijing, China). GH3 cells were cultured in Ham’s F12 medium supplemented with 10% FBS and 1% streptomycin and penicillin in a humidified 5% CO_2_ incubator at 37°C.

A pWPT lentiviral expression vector (Genechem, Shanghai, China) was used to clone wild-type and mutant *GNAS*, and the generated constructs were termed pWPT-GNAS (expressing *GNAS* wild-type), pWPT-GNAS-Q227L (expressing *GNAS* mutated at Q227L), and pWPT-GNAS-R201C (expressing *GNAS* mutated at R201C). GH3 cells were plated in 6-well plates at 70% confluence and then injected with lentivirus to express wild-type or mutant-type *GNAS*. Overexpressed or knocked-down *MEG3* gene was packaged by lentivirus and then transfected into GH3 cells according to the protocols (Corues Biotechnology, Nanjing, China).

### RNA extraction and quantitative reverse transcription real-time quantitative PCR

Total RNA was isolated from tissues or cells using Trizol reagent and reverse transcribed into complementary DNA using the TaqMan MicroRNA Reverse Transcription Kit (TaKaRa, Dalian, China). Real-time quantitative (RT-q) PCR was performed using SYBR Green PCR Master Mix (Takara, Japan) according to the manufacturer’s instructions. The sequences of qPCR primers are listed in Table 2.

lncRNA *MEG3* was normalized with β-actin and the level of *MEG3* in GHPA was further normalized with its level in NFPA. The experiments were repeated at least three times.

### RNA-Seq analysis

Total RNA from GH3 cells with MEG3-overexpressed and MEG3-overexpress-vector was extracted using Trizol reagent (Thermo Fisher, Waltham, USA) according to the manufacturer’s protocol. RNA samples were digested with RNase free DNase I (Invitrogen, Carlsbad, USA) to eliminate residual genomic DNA, and the digestion products were purified using magnetic beads (Axygen, Union City, USA).

Total RNA was qualified by Agilent 2100 Bioanalyzer (Agilent Technologies, Palo Alto, CA, USA). The next-generation sequencing library was prepared according to the protocol provided by the manufacturer (NEBNext Ultra RNA Library Prep Kit for Illumina HiSeq system). The sequences and data were analyzed to identify differentially expressed genes (Genewiz, Shanghai, China).

### Western blotting

Proteins were extracted from tumor cells and tissues in RIPA buffer (Beyotime Biotech., Shanghai, China), separated using SDS-PAGE, and transferred onto polyvinylidene fluoride membranes. The membranes were blocked with 5% fat-free milk in Tris-buffered saline containing 0.1% Tween 20 and incubated at 4°C overnight with primary antibodies against MMP-2 (#40994, rabbit mAb, 1:1000), β-catenin (#8480, rabbit mAb, 1:1000), MMP-9 (#13667, rabbit mAb, 1:1000), and β-actin (#4970, rabbit mAb, 1:1000), purchased from Cell Signaling Technology (Danvers, MA, USA). Subsequently, the membranes were incubated with horseradish peroxidase-conjugated secondary antibodies (#7074, anti-rabbit IgG, HRP-linked antibody, Cell Signaling Tech.). The images were visualized using enhanced chemiluminescence (Beyotime Biotech., Shanghai, China). Pierce ECL Western Blotting Substrate (Thermo Scientific) was used to detect the chemiluminescence signals. Densitometric analysis of the western blot bands was performed using the Bio-Rad Imaging system (Bio-Rad, Hercules, California, USA). The western blotting assay was repeated three times.

### Transwell assay

Matrigel matrix diluent (300 μg/mL) was used to coat the bottom of the upper chamber of the Transwell chamber in a precooled environment at 4°C. The volume ratio of serum-free F12 medium to Matrigel in the upper chamber was 4:1, the total volume was 100 μL, and the Matrigel was frozen overnight at 4°C. The next day, 500 μL of F12 medium containing 10% FBS was added into the lower chamber. Cells (1 × 10^5^) were seeded in the upper chamber. After incubation for 48 h, non-invading cells in the upper chamber were removed. Cells that invaded the bottom chamber were fixed with 4% paraformaldehyde and stained using 0.1% crystal violet (Beyotime Biotech., Shanghai, China). Five random fields were selected for counting under a microscope (magnification, ×200). Each experiment was performed three times.

### Immunofluorescence

Tumor tissue sections were prepared from the surgical resection of GHPA. Tissue slides were fixed with 4% formaldehyde, blocked with 10% normal goat serum, and incubated overnight at 4°C with primary antibodies against β-catenin, E-cadherin, N-cadherin, and vimentin (diluted at 1:100 in PBS). All antibodies were obtained from Cell Signaling Technology (Danvers, MA, USA). The slides were removed from the incubation chamber, washed three times with PBST, and incubated for 40 min in the dark in a humidified chamber at room temperature with goat polyclonal secondary antibodies against rabbit IgG (1:300; Abcam Biotechnology, Cambridge, MA, USA) reconstituted in PBS. Sections were washed three times with PBST. After counterstaining was complete, the sections were treated with glycerol/PBS (2:1) for 10 min in the dark at room temperature. Five randomly chosen fields were selected for counting using an Axiovert 200 fluorescent microscope.

### Subcutaneous xenografts in nude mice

Animal experiments were approved by the Animal Experimentation Ethics Committee of the Jinling Hospital of Nanjing University (protocol number: 2021DZGKJDWSL-0081).

Four-week-old female athymic BALB/c nude mice were purchased from the Shanghai SLAC Laboratory Animal Co., Ltd. (Shanghai, China) and housed and maintained in laminar airflow chambers under specific pathogen-free conditions. *MEG3*-overexpressing GH3 cells (10^7^ cells/0.1 mL) were subcutaneously injected into the right back side of the mice. Following the tumor formation, a week after injection, lithium chloride (Sigma-Aldrich, St. Louis, MO, USA; 60 mg/kg/d in 100 μL saline), a β-catenin activator, was administered daily via intraperitoneal injection in the group with high levels of *MEG3*. The other groups were injected with saline alone as controls. Tumor volumes were measured using a vernier caliper twice a week and calculated as (length × width^2^)/2. Four weeks after the injection, the mice were euthanized via cervical dislocation, and the dissected tumors were weighed and processed to determine the expression levels of related proteins, including β-catenin, E-cadherin, N-cadherin, MMP-2, and MMP-9.

### Immunohistochemistry

Tumor tissues from mice were fixed and antigens were exposed using heat-induced epitope retrieval with exposure to citric acid buffer (pH = 7.0), blocked in 10% normal goat serum, and incubated with 3% hydrogen peroxide. The tissue slides were incubated with primary antibodies against β-catenin (#8480, 1:200), E-cadherin (#3195, 1:200), N-cadherin (#13116, 1:200), MMP-2 (#40994, 1:200), and MMP-9 (#13667, 1:200) from Cell Signaling Technology (Danvers, MA, USA) overnight at 4°C followed by incubation with a goat anti‑rabbit secondary antibody (A0208, 1:50) from Beyotime for 15 min at 37°C. We repeated the experiments thrice. The number of positive cells was counted in each section in 10 randomly selected fields (magnification, ×200).

### Statistical analysis

Data were statistically analyzed using SPSS 19.0. Student’s unpaired *t*-test and Fisher’s exact test were used for intergroup analysis. One‑way analysis of variance test was used to compare tumor volume and weight among the three groups of *in vivo* experiments when the mice were euthanized. The results are presented as mean ± standard deviation. Correlation was analyzed using Spearman’s correlation. *p* < 0.05 was considered statistically significant.

## Results

### *GNAS* mutations in GHPA

In total, 44 patients with GHPA and 10 patients with NFPA were enrolled in this study. Mutations in *GNAS* were scanned by directly sequencing genomic PCR products amplified from the tumor samples of patients using specific primer sets. The results revealed that 16/44 patients carried *GNAS* mutations, including 8 cases of codons 227 and 201 each. Conversely, no *GNAS* mutation was detected in the 10 patients with NFPA. Subsequently, the patients were divided into two groups (GNAS-WT, n = 28; GNAS-MUT, n = 16). The percentage of invasive tumors (Knosp grades 3 and 4) in GNAS-MUT tumors remarkably decreased (18.7% *vs*. 71.4%; *p* = 0.001; Table 1). There were no statistical differences in terms of age or gender between the two groups.

### Mutant *GNAS* leads to the inhibition of GH3 cell invasion

**Figure 1:**
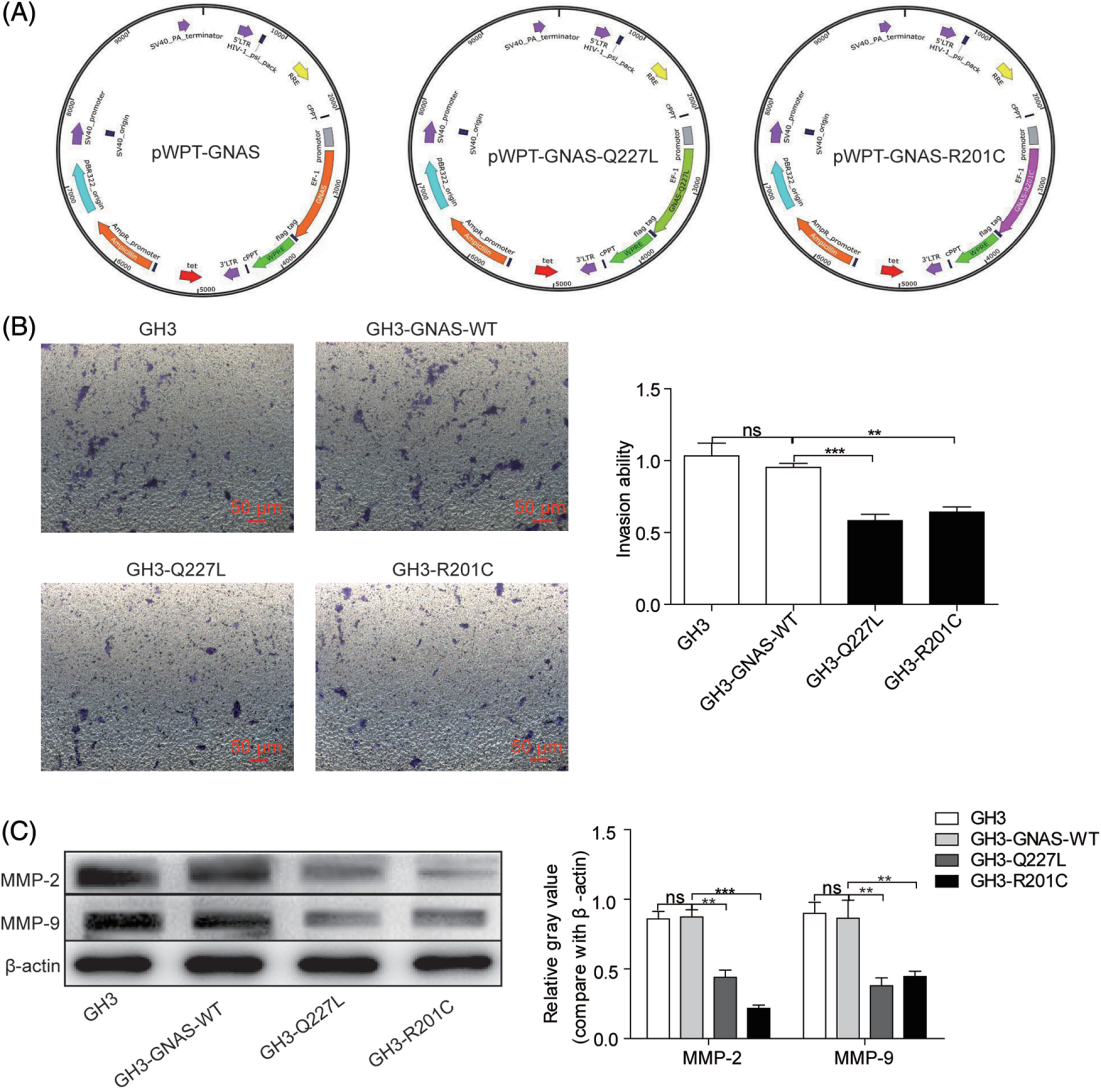
*GNAS* mutations inhibit GH3 cell invasion. A: Wild-type and mutant *GNAS* were expressed in GH3 cells by transduction with lentiviral vectors: pWPT-GNAS-Q227L, pWPT-GNAS-R201C, and pWPT-GNAS. B: Cell invasion was measured using the Transwell assay (magnification, ×200). C: The levels of MMP-2 and MMP-9 were quantified via western blotting. **p < 0.01, ***p < 0.001 show statistical significance between the two groups as indicated, and “ns” shows no significance. GH3-GNAS-WT: GH3 cells containing wild-type *GNAS*; GH3-Q227L: GH3 cells expressing the mutant *GNAS* at Q227L; GH3-R201C: GH3 cells expressing the mutant *GNAS* at R201C. Data represent the mean ± s.d. of three independent experiments

The clinical analysis results predicted that *GNAS* mutations are associated with the invasiveness of GHPA. No *GNAS* mutations were detected in GH3 cells. Accordingly, GH3 cells expressing the wild- or mutant-type *GNAS* were constructed. The cells were injected with lentivirus carrying Flag-tagged Q227L (pWPT-GNAS-Q227L) or R201C (pWPT-GNAS-R201C) as well as GNAS-WT control (pWPT-GNAS) (Fig. 1A). The cell invasion capacity was significantly decreased in GH3-Q227L (*p* = 0.008) and GH3-R201C (*p* = 0.002) cells compared with GH3-GNAS-WT cells (Fig. 1B). In addition, the expression levels of MMP-2 (2-fold, *p* = 0.0015; 4-fold, *p* = 0.0003) and MMP-9 (2.3-fold, *p* = 0.0025; 1.9-fold, *p* = 0.0036) in GH3-Q227L and GH3-R201C cells were reduced (Fig. 1C).

### Mutant *GNAS* upregulates *MEG3* expression

**Figure 2:**
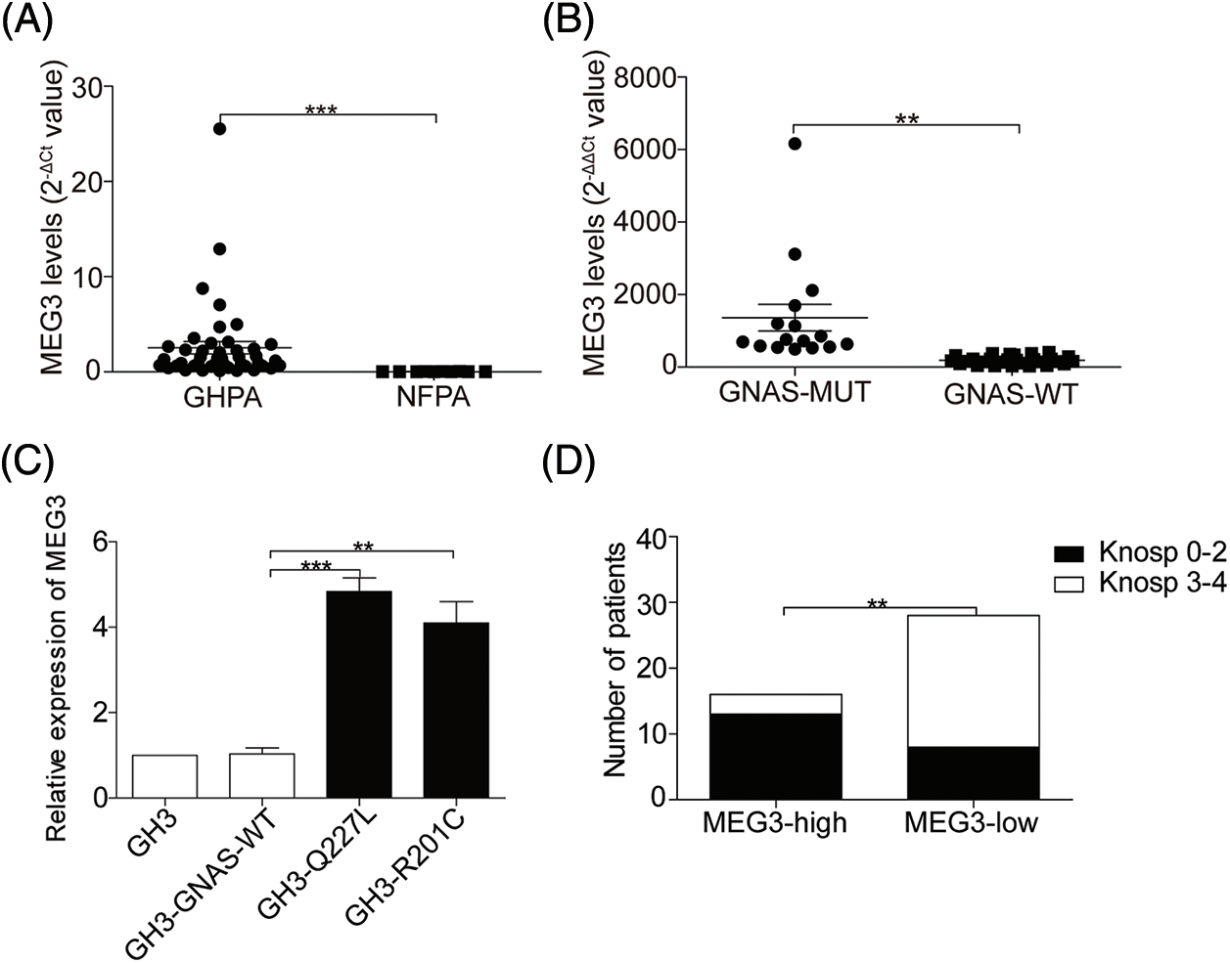
*GNAS* mutations upregulate *MEG3* expression. A: The expression of *MEG3* in *GHPA* and NFPA was quantified via RT-qPCR analysis. B and C: Correlation between *MEG3* expression and *GNAS* mutations was determined via RT-qPCR. D: Correlation between *MEG3* expression and percentage of invasive tumors was analyzed. **p < 0.01, ***p < 0.001 show statistical significances between the two groups as indicated. GHPA: growth hormone–secreting pituitary adenoma; NFPA: non‑functioning pituitary adenoma; GNAS-MUT: growth hormone–secreting pituitary adenoma possessing *GNAS* mutations; GNAS-WT: growth hormone–secreting pituitary adenoma possessing wild-type *GNAS*; MEG3-high and MEG3-low: GHPAs were divided into the *MEG3* high expression group and low expression group according to the *MEG3* expression level

Because *GNAS* mutations are speculated to participate in upregulating *MEG3* expression, the expression levels of *MEG3* in NFPA and GHPA tumor tissues were quantified via RT-qPCR. The levels of *MEG3* considerably increased in 44 GHPA tumor tissues compared with the 10 NFPA tumor tissues (*p* = 0.0003) (Fig. 2A). Intriguingly, *MEG3* expression further increased in the 16 GHPA tumor tissues with mutant *GNAS* compared with the 28 wild-type tumors (*p* = 0.006; Fig. 2B). Consistently, the high levels of *MEG3* were verified in GHPA tumor tissues with single mutant sites at Q227L and R201C (4.7-fold, *p* = 0.0004; 4.4-fold, *p* = 0.0033; Fig. 2C). Importantly, tumor invasiveness significantly declined in the group expressing high levels of *MEG3* compared with the group expressing low levels of *MEG3* (*p* = 0.0014; Fig. 2D). We suggest that *MEG3* negatively correlates with tumor invasion in GHPA.

### *MEG3* inhibits the invasiveness of GH3 cells

**Figure 3:**
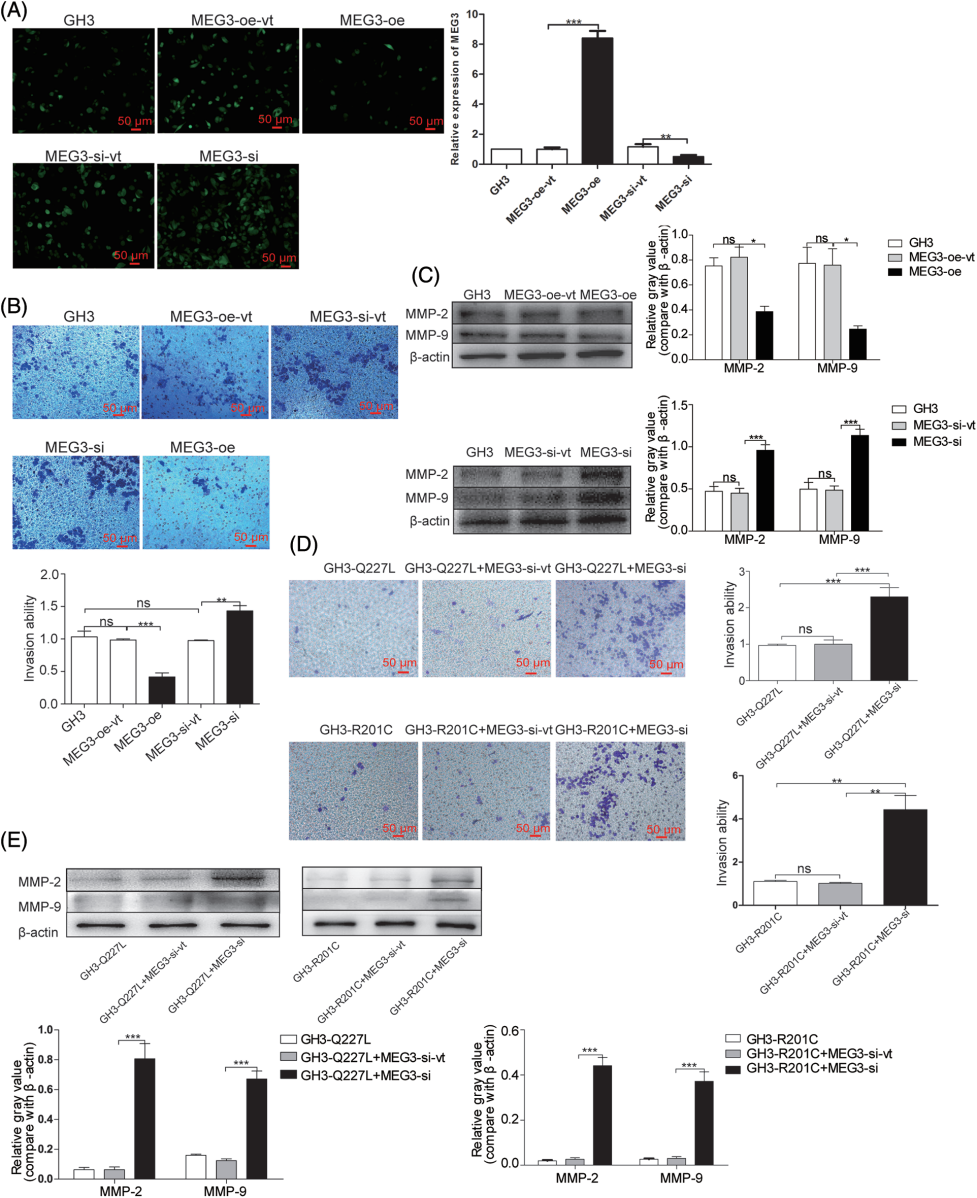
*MEG3* inhibits the invasiveness of GHPA cells. A: *MEG3* was overexpressed or knocked down in GH3 cells. Relative levels of *MEG3* were quantified via immunofluorescence assay and confirmed via RT-qPCR (magnification, ×200). B: Cell invasion was analyzed using the Transwell assay (magnification, ×200). C: Expression levels of MMP-2 and MMP-9 in *MEG3*-overexpressing cells were measured via western blotting. D: Cell invasion of *MEG3* knockdown in GH3 cells expressing mutant *GNAS* was detected using the Transwell assay (magnification, ×200). E: Expression levels of MMP-2 and MMP-9 of *MEG3* knockdown in GH3 cells expressing mutant *GNAS* were measured via western blotting. *p < 0.05, **p < 0.01, and ***p < 0.001 show the statistical significance between the two groups as indicated, and “ns” shows no significance. *MEG3*-oe-vt: GH3 cells expressing empty vector of overexpressed *MEG3*; *MEG3*-oe: GH3 cells expressing overexpressed *MEG3*; *MEG3*-si-vt: GH3 cells containing *MEG3* siRNA expressing empty vector; *MEG3*-si: GH3 cells containing *MEG3* siRNA expressing vector

To verify whether *MEG3* can inhibit rat GH3 cell invasion, *MEG3* was manipulated using lentiviral expression systems for ectopic expression or knock down in GH3 cells. After lentivirus infection, *MEG3* was quantified using immunofluorescence and RT-qPCR. *MEG3* level increased >8-fold in MEG3-overexpressing cells compared with MEG3-overexpressing empty vector cells (*p* = 0.0001); *MEG3* level decreased >2-fold in MEG3-si cells (*p* = 0.005) (Fig. 3A). Compared to the lentiviral vector control, *MEG3* overexpression decreased cell invasion (*p* = 0.0008). By contrast, silencing *MEG3* increased cell invasion (*p* = 0.0044) (Fig. 3B). Consistently, protein expression of MMP-2 (*p* < 0.05) and MMP-9 (*p* < 0.05) decreased in MEG3-overexpressing cells and increased in cells with *MEG3* silenced (*p* < 0.001, *p* < 0.001) (Fig. 3C). To evaluate the role of *MEG3* in mediating the effects of the *GNAS* mutation on tumor invasion, we examined the effects of *MEG3* knockdown in GH3 cells expressing mutant *GNAS*. Cell invasion ability and protein levels of MMP-2 and MMP-9 significantly increased in GH3 cells with *GNAS* mutations and *MEG3* knockdown (Figs. 3D and 3E). These results suggested that *GNAS* mutations inhibit invasiveness of rat GHPA cells by activating *MEG3*.

### *MEG3* inhibits cell invasion by inactivating the Wnt/β-catenin signaling pathway

**Figure 4:**
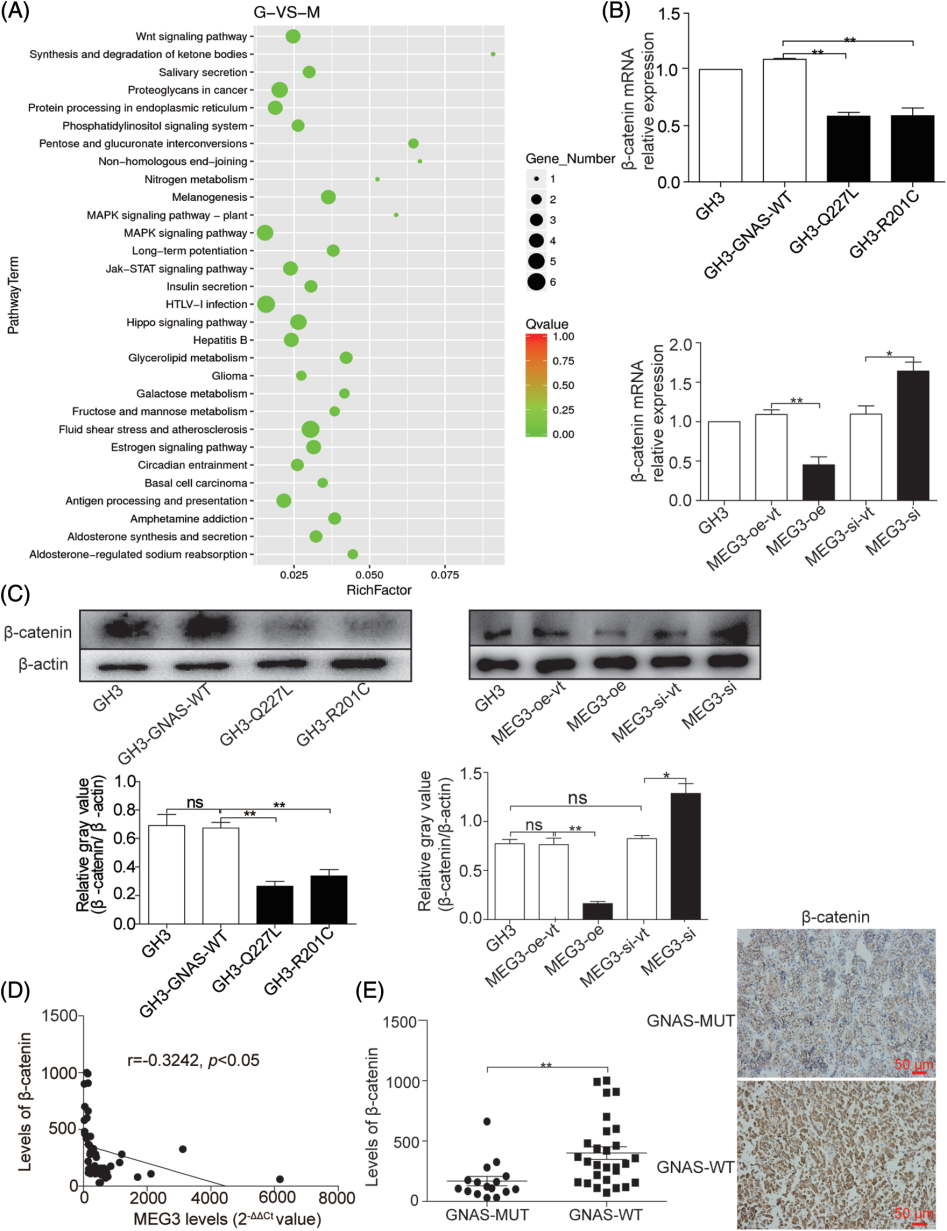
*MEG3* inhibits the invasiveness of GHPA with *GNAS* mutations by inactivating Wnt/β-catenin signaling. A: RNA-Seq was used to examine the gene expression profiles in *MEG3*-overexpressed GH3 cells compared with vector-only controls. The altered mRNA expression profile was analyzed using Kyoto Encyclopedia of Genes and Genomes pathway enrichment analysis. B: Reduction of β-catenin mRNA expression in GHPA tumors with the *GNAS* mutations and GH3 cells with a high level of *MEG3* was confirmed via RT-qPCR. C: Relative β-catenin levels were measured via western blotting. D: Significant negative relationships were found between the expression levels of *MEG3* and β-catenin. E: β-catenin levels between *GNAS*-mutant and wild-type tumors were analyzed using IHC (magnification, ×200). *p < 0.05 and **p < 0.01 show the statistical significance between the two groups as indicated, and “ns” shows no significance

To investigate the mechanism by which *MEG3* inhibits GHPA cell invasion, *MEG3* was overexpressed in GH3 cells and the RNA expression profile was analyzed by RNA-Seq. The results revealed that the Wnt/β-catenin signaling pathway could be involved in regulating cell invasion (Fig. 4A). Consistently, the mRNA level of β-catenin significantly decreased in GH3 cells with *GNAS* mutations (GH3-Q227L: *p* = 0.0047, GH3-R201C: *p* = 0.005) (Fig. 4B). Ectopic expression of *MEG3* in GH3 cells resulted in reduced β-catenin expression (*p* < 0.01); conversely, silencing of *MEG3* in GH3 cells increased the levels of β-catenin (*p* < 0.05) (Fig. 4B and C). Significant negative relationships were found between the expression levels of *MEG3* and β-catenin (*p* < 0.05) (Fig. 4D). Consistently, the level of β-catenin in GHPA-carrying *GNAS* mutations (n = 16) was lower than that in wild-type tumors (n = 28) (*p* = 0.001), which are associated with high levels of *MEG3* (Fig. 4E). The results suggest that *MEG3* negatively regulates β-catenin and promotes cell invasion, particularly in GHPA cells carrying *GNAS* mutations.

### Inactivation of β-catenin suppresses GHPA cell invasion

**Figure 5:**
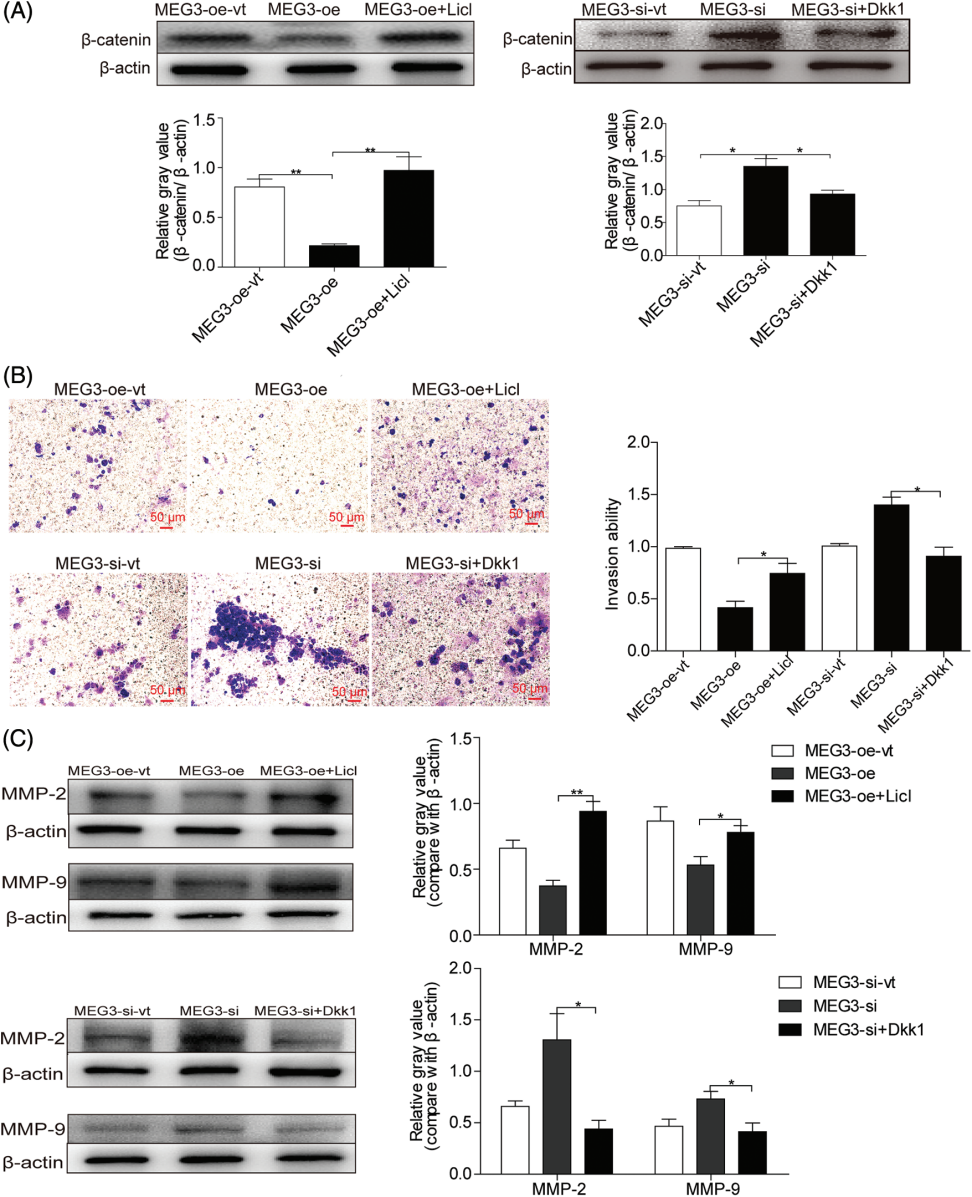
*MEG3*-mediated downregulation of β-catenin inhibits GH3 cell invasion. A: *MEG3* was manipulated by expressing or silencing in GH3 cells. The expression of β-catenin was induced by LiCl (20 mM) or repressed by Dkk1 (150 ng/mL) and measured using western blotting. B: Relative cell invasion was quantified by the Transwell assay (magnification, ×200). C: Expression of MMP-2 and MMP-9 was measured using western blotting. *p < 0.05, **p < 0.01 show statistical significance between the two groups as indicated

LiCl is an activator and Dickkopf1 (Dkk1) is a suppressor of β-catenin [24,25]. Accordingly, MEG3-overexpressing cells were treated with 20 mM LiCl [26] and MEG3-si cells were treated with 150 ng/mL Dkk1 [27]. β-catenin expression increased in LiCl-treated cells (4.4-fold, *p* < 0.01) but decreased in Dkk1-treated (Sigma-Aldrich) cells (1.4-fold, *p* < 0.05; Fig. 5A). LiCl treatment enhanced cell invasion (1.8-fold, *p* < 0.05); however, Dkk1 treatment inhibited cell invasion (1.6-fold, *p* < 0.05; Fig. 5B). LiCl could upregulate the expression of MMP-2 (*p* < 0.01) and MMP-9 (*p* < 0.05). Conversely, Dkk1 suppressed MMP-2 (*p* < 0.05) and MMP-9 (*p* < 0.05) expression (Fig. 5C). These results suggest that *MEG3* suppresses GHPA cell invasion by inhibiting activated β-catenin.

### *GNAS* mutations inhibit EMT

**Figure 6:**
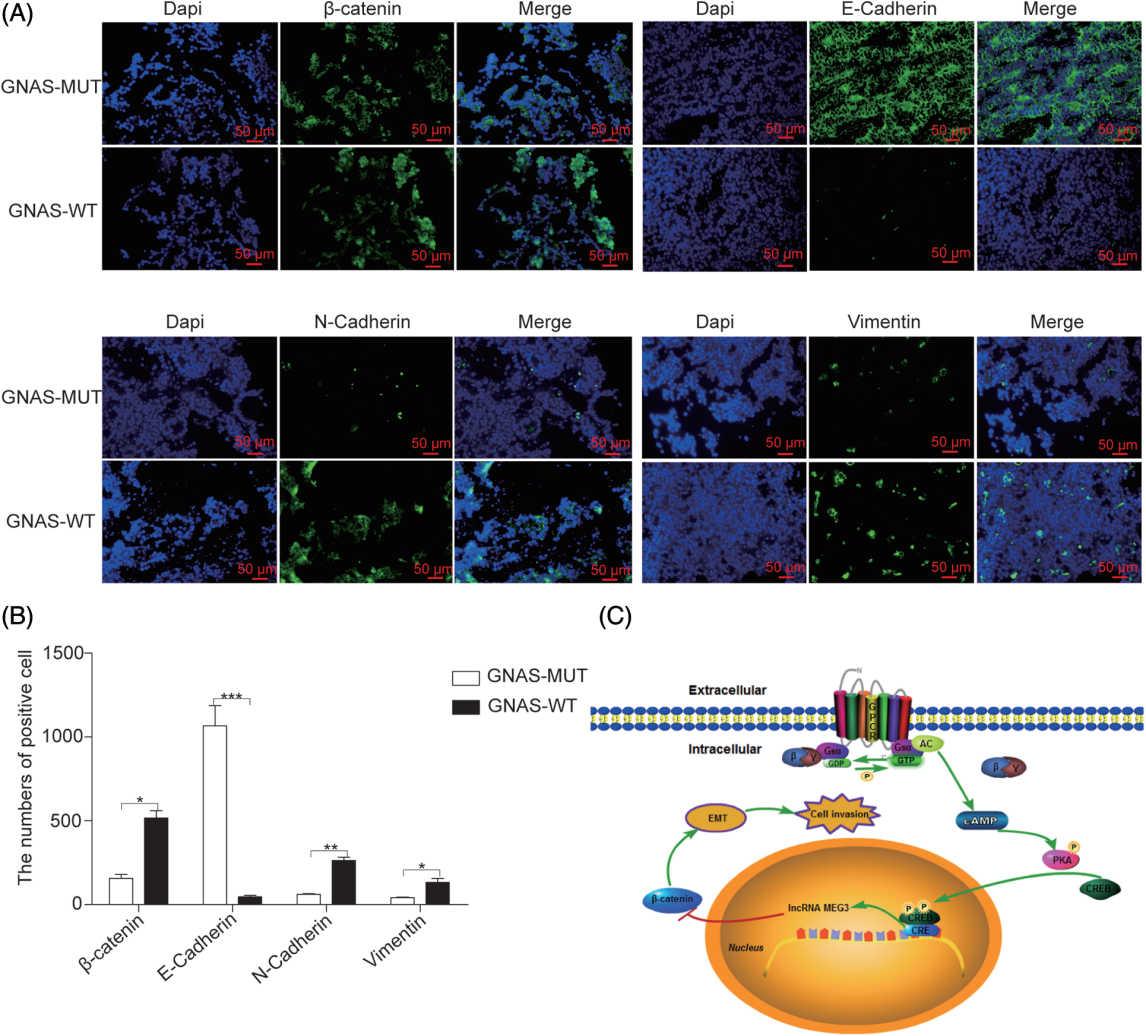
*GNAS* mutations inhibit EMT in GHPA tumors. A and B: The expression of EMT-related proteins in GHPA tumor tissues was quantified via immunofluorescence (magnification, ×200). C: Mechanism by which *GNAS* mutations inhibit the invasiveness of GHPA. *p < 0.05, **p < 0.01, and ***p < 0.001 show statistical significance between the two groups as indicated. GPCR: G protein coupled receptor; Gs_α_: α subunit of Gs protein; GDP: guanosine diphosphate; GTP: guanosine triphosphate; AC: adenylyl cyclase; cAMP: cyclic adenosine monophosphate; PKA: protein kinase A; CREB: cAMP response element-binding protein; CRE: cAMP response element; EMT: epithelial–mesenchymal transition

EMT plays a fundamental role in promoting cell mobility and tumor metastasis, and Wnt/β-catenin signaling is a key mechanism in EMT [28,29]. To examine the effects of *GNAS* mutations on EMT, we quantified the expression of EMT-associated proteins regulated by β-catenin, such as E-cadherin (cell adhesion marker), as well as N-cadherin and vimentin (mesenchymal markers), using immunofluorescence in GHPA tissues. Compared with GHPA tissue with wild-type *GNAS*, GHPA tissue with *GNAS* mutations exhibited increased E-cadherin (*p* < 0.0001), decreased N-cadherin (*p* < 0.01) and vimentin (*p* < 0.05), and high constitutive levels of *MEG3* (Fig. 6A and B). Thus, our findings suggest that *GNAS* mutations inhibit the invasiveness of GHPA by downregulating EMT (Fig. 6C).

### In vivo validation of *MEG3*-mediated inhibition of cell invasion

**Figure 7:**
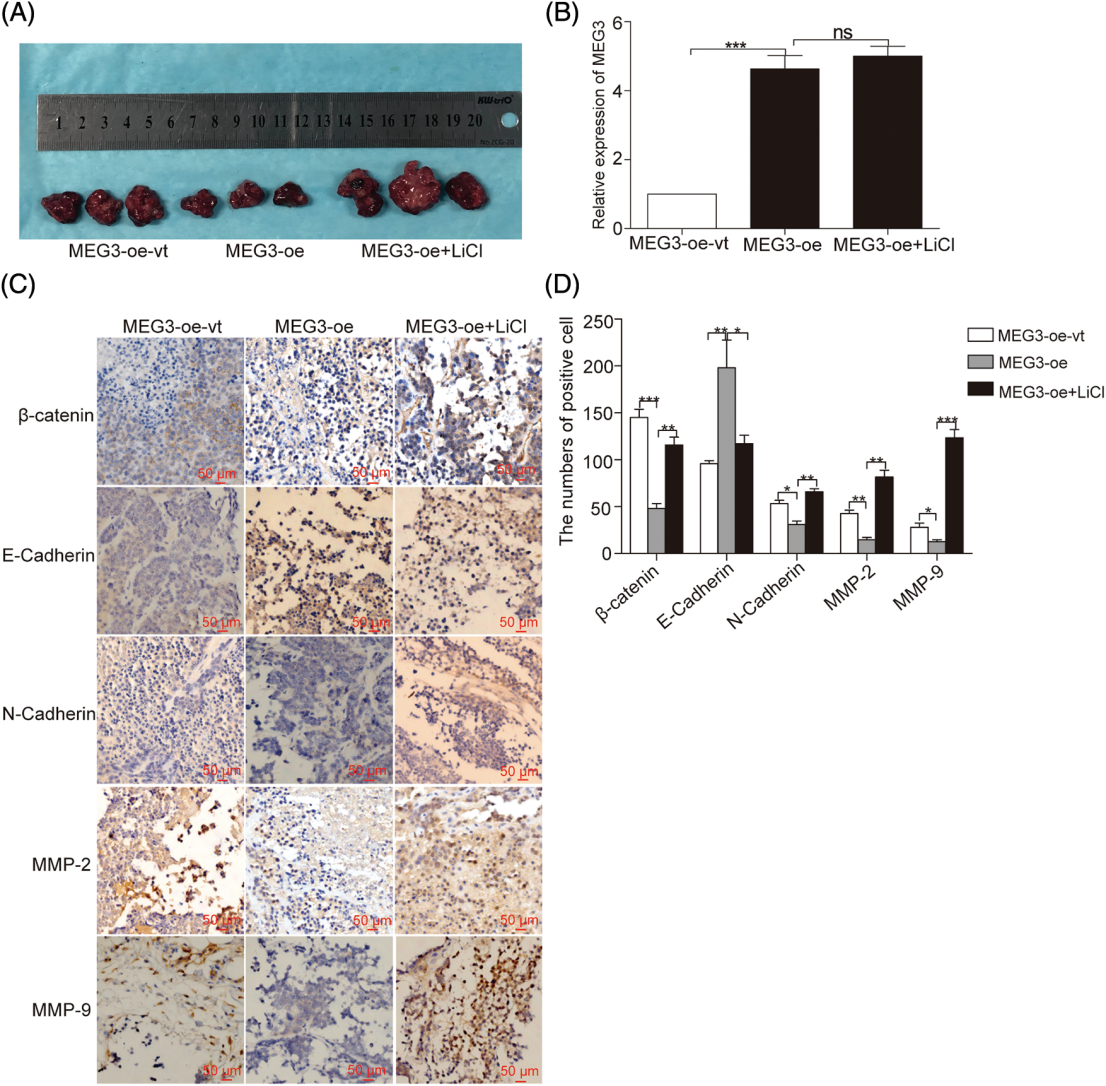
*MEG3* inhibits GHPA cell invasion *in vivo*. A: GH3 cells expressing different levels of *MEG3* were subcutaneously injected into null mice followed by LiCl treatment. B: Sustained efficiency of *MEG3* upregulation was verified by detecting the expression level of *MEG3* in the xenograft tumor of each group via RT-qPCR. C, D: Levels of β-catenin, E-cadherin, N-cadherin, MMP-2, and MMP-9 in tumor tissues were quantified via immunohistochemistry (IHC) (magnification, ×200). *p < 0.05, **p < 0.01, and ***p < 0.001 show statistical significance among the three groups as indicated, and “ns” shows no significance

To further verify that *MEG3* suppresses cell invasion in GHPA by inhibiting β-catenin-regulated EMT, β-catenin was manipulated by overexpressing *MEG3* in GH3 cells. The cells were subcutaneously injected into nude mice for tumor formation (Fig. 7A). The sustained efficiency of *MEG3* upregulation was verified by detecting the expression level of *MEG3* in the xenograft tumor of each mouse via RT-qPCR. Notably, *MEG3* level increased >four-fold in the *MEG3*-overexpressing group compared with the *MEG3*-oe-vt group and was unaffected by LiCl (Fig. 7B). Consistent with the regulated levels of β-catenin in the formed tumors, E-cadherin increased upon overexpressing *MEG3* and further decreased following LiCl treatment. Conversely, the expression levels of N-cadherin, β-catenin, MMP-2, and MMP-9 decreased by elevating *MEG3* and increased via LiCl-mediated induction (Figs. 7C and 7D). The results confirmed that *MEG3* negatively regulates EMT by downregulating β-catenin.

## Discussion

GHPA is a typically benign tumor with a high incidence and large economic burden and often manifests with invasive growth [30]. Numerous studies have indicated that the presence of point mutations in *GNAS* strongly reflects the biological characteristics of GHPAs, such as tendency for densely granulated tumors and smaller tumor size [31–33]. A meta-analysis has suggested that *GNAS* mutations can be used as a prognostic factor for treatment response to somatostatin analogs [34]. A study has suggested that Gsα protein encoded by *GNAS* is important for activating the cAMP-dependent pathway in pituitary cells for differentiation and proliferation [35]. *GNAS* mutations were proposed to be involved in the constitutive activation of cAMP, which plays a causal role in pituitary adenomas [36]. However, the poor growth rate of GHPAs naturally expressing *GNAS* mutations strongly suggest counteraction of the putative growth advantage conferred by cAMP. Consequently, this study aimed to elucidate the effect of *GNAS* mutations on the GHPA phenotype, especially on tumor invasion, and the mechanism involved.

Interestingly, *MEG3* was identified as a tumor suppressor that is a downstream target of cAMP [12,37,38]. Studies have reported that the level of *MEG3* is uniquely high in GHPA but not in NFPAs [39]. Thus, we speculate that *GNAS* mutations suppress the invasiveness of GHPA mainly by activating *MEG3*. As expected, the high levels of *MEG3* were only detected in GHPA-carrying *GNAS* mutations. *MEG3* levels were significantly increased in GH3 cells expressing *GNAS* mutations compared with GH3 cells expressing wild-type *GNAS*, suggesting that *GNAS* mutations inhibit GHPA cell invasion through *MEG3* activation. To ascertain the effect of *MEG3* on cell invasion, we manipulated *MEG3* in GH3 cells. Ectopic expression of *MEG3* reduced cell invasion, and conversely, silencing *MEG3* enhanced invasiveness. Altogether, our results suggest that *MEG3* plays an important role in promoting GHPA invasiveness.

The canonical Wnt/β-catenin signaling pathway is a key regulator of EMT and tumor progression [40]. Upon activation of the Wnt pathway, β-catenin accumulates in nuclei and leads to EMT [17]. In this study, we showed that *GNAS* mutations lead to increased *MEG3* expression but decreased β-catenin expression. The activation of β-catenin by LiCl enhanced cell invasion, and the inactivation of β-catenin by Dkk1 inhibited cell invasion. Subsequently, elevated *MEG3* expression led to the downregulation of β-catenin in GHPA cells. In parallel, the silencing of *MEG3* upregulated β-catenin expression, suggesting that *MEG3* inhibits the invasiveness of GHPA cells by inactivating the Wnt/β-catenin pathway. However, in this study, the mechanism by which *MEG3* regulates β-catenin is unclear. Recent studies have shown that *MEG3* inhibits pituitary tumor invasiveness through the *MIR-376B-3P/HMGA2* axis [41]. Other studies have shown that *MEG3* negatively regulates the proliferation, migration, and apoptosis of osteosarcoma through the Wnt/β-catenin pathway by targeting miR-184 [42]. *MEG3* suppressed liver cancer cell growth by inhibiting β-catenin through the activation of *PKM2* and inactivation of *PTEN* [15]. The mechanism by which *MEG3* regulates β-catenin-mediated transcriptional activation in GHPA must be investigated.

Anterior pituitary with an epithelial phenotype expresses multiple cadherin proteins, such as E-cadherin, that function in cell attachment [43]. EMT is vital for tumor cell invasion and metastasis. The loss of E-cadherin and/or the increase of N-cadherin are hallmarks of EMT. In various tumor types, the Wnt/β-catenin signaling pathway is constitutively active to promote EMT [44]. This study uncovered that β-catenin and EMT-related functional proteins are altered by *MEG3* in GHPA with *GNAS* mutations. Accordingly, overexpressing *MEG3* in GHPA cells led to the upregulation of E-cadherin but downregulation of N-cadherin and vimentin by altering the transcriptional regulation of β-catenin.

MMPs—important proteolytic enzymes in the degradation of extracellular matrix and basement membrane—are crucial for tumor cell invasion [45]. MMP-2 and MMP-9 play vital roles in tumor invasion due to their potent ability to degrade collagen type IV [46]. In particular, they play important roles in the invasiveness of GHPA [47]. Consistently, in this study, the levels of MMP-2 and MMP-9 decreased in GHPA tumors with *GNAS* mutations; additionally, the increase in *MEG3* expression in GHPA cells resulted in a decrease in the expression of MMP-2 and MMP-9.

Of note, our findings identify a possible mechanism to explain several known GHPAs phenotypes. Our observations that *MEG3* activation in somatotroph cells due to an activating *GNAS* mutation, concomitantly the inactivation of Wnt/β-catenin pathway and inhibition of EMT may explain distinctive somatotroph adenoma features. However, evidence from other studies suggested that somatotroph cAMP has a dual role in promoting growth hormone secretion while causing DNA damage [35]. These findings may help explain the smaller, less invasive size of GHPAs containing *GNAS* mutations, both of which secrete higher levels of growth hormone. Likewise, in our present study, we speculate that cAMP also plays a dual role in *GNAS* mutation–positive adenomas. The different mechanism is that *MEG3* may also play an important role in inhibiting tumor proliferation and invasion in addition to DNA damage.

## Conclusions

This study revealed that *GNAS* mutations are associated with the invasiveness of GHPA tumors, possibly by increasing the level of *MEG3*. *MEG3* upregulation in rat GH3 cells suppress cell invasion by inhibiting Wnt/β-catenin signaling. Silencing *MEG3* upregulates β-catenin and enhances EMT. Owing to the lack of growth advantage observed in GHPAs with mutant *GNAS*, the possible mechanisms in this study were elucidated. Moreover, the findings suggest that *MEG3* serves as a biomarker for detecting the GHPA phenotype and inhibition of Wnt/β-catenin signaling can be a useful approach for treating GHPA.

## Data Availability

The datasets during and analyzed during the current study are available from the corresponding author on reasonable request.

## Abbreviations

GHPA: Growth hormone–secreting pituitary adenoma
NFPA: nonfunctioning pituitary adenoma
*GNAS*: α subunit of the stimulatory G protein
*MEG3*: maternally expressed 3
EMT: epithelial-to-mesenchymal transition
CCK-8: Cell Counting Kit-8 (tradename)
MMP: matrix metalloproteinase
siRNA: small interfering RNA or short interfering RNA
WT: wild type
AC: adenylyl cyclase
cAMP: Cyclic 3′,5′-adenosine monophosphate
